# Early time-of-day infusion of PD-1 inhibitor improves prognosis in advanced gastric cancer: a multicenter real-world study

**DOI:** 10.3389/fimmu.2026.1945065

**Published:** 2026-09-18

**Authors:** Ziting Qu, Xiaoyu Guo, Shupei Zhang, Yanyan Zhao, Zhenyan Hu, Wenxi Ding, Sachiyo Nomura, Yiyin Zhang, Xiaoli Wei

**Affiliations:** 1 Department of Oncology, the First Affiliated Hospital of Anhui Medical University, Hefei, China; 2 Department of Oncology, the Second Affiliated Hospital of Anhui Medical University, Hefei, China; 3 Department of Oncology, the Second Affiliated Hospital of Bengbu Medical University, Bengbu, China; 4 Department of Medicine II, Section Molecular Hepatology, University Medical Center Mannheim, Medical Faculty Mannheim, Heidelberg University, Mannheim, Germany; 5 Department of Gastrointestinal Surgery, Graduate School of Medicine, The University of Tokyo, Tokyo, Japan; 6 Department of Pharmacology, Toxicology and Therapeutics, the University of Kansas Medical Center, Kansas, KS, United States

**Keywords:** chrono-immunology, gastric cancer, immune checkpoint inhibitors, prognosis, time of day of administration

## Abstract

**Background:**

Whether the time-of-day administration (ToDA) of programmed cell death 1 (PD-1) inhibitor infusion influences survival in advanced gastric cancer (AGC) remains unclear. This research aimed to evaluate the impact of ToDA on the prognosis of AGC patients.

**Methods:**

This multicenter retrospective study enrolled 416 AGC patients receiving PD-1 inhibitor therapy, who were divided into early and late ToDA groups based on sensitivity analyses using 30-minute intervals of the administration time window. After 1:2 propensity score matching (PSM), progression-free survival (PFS) and overall survival (OS) were compared between groups, and subgroup analyses were conducted. Subsequently, prognostic risk score models were established based on independent prognostic factors.

**Results:**

A cutoff time of 15:00 was established via sensitivity analyses in the overall cohort. Patients were categorized into early and late ToDA groups. After PSM, multivariable Cox analyses identified late ToDA as an independent risk factor for both PFS (*P* < 0.001) and OS (*P* = 0.004). Kaplan–Meier curves demonstrated significantly better PFS (*P* = 0.003) and OS (*P* = 0.003) for the early ToDA group. In the overall cohort, prognostic risk score models incorporating ToDA, metastasis sites, treatment lines, neutrophil-to-lymphocyte ratio (NLR), human epidermal growth factor receptor 2 (HER-2) status and programmed cell death ligand 1 (PD-L1) status effectively stratified patients into low-, middle-, and high-risk groups with distinct survival (*P* < 0.001). The detrimental effect was observed across most major subgroups, though several subgroup analyses were not statistically significant.

**Conclusions:**

This research provides real-world evidence that early ToDA of PD-1 inhibitors is associated with improved prognosis in AGC patients, providing a rationale for chronotherapy optimization and introducing a preliminary risk stratification model for AGC immunotherapy. The risk scoring model requires prospective validation.

## 1 Introduction

Gastric cancer remains a leading cause of cancer-related mortality, and patients with advanced gastric cancer (AGC) face a dismal prognosis (1, 2). The introduction of immune checkpoint inhibitors (ICIs), particularly programmed death-1 (PD-1) inhibitors, has revolutionized the therapeutic landscape for AGC (3–5). However, responses to PD-1 inhibitor therapy remain highly heterogeneous in clinical practice (6). Consequently, identifying strategies to optimize immunotherapy efficacy and improve patient survival remains an urgent priority.

Circadian rhythms have a significant impact on the distribution and function of immune cells and are also key factors influencing the efficacy of immunotherapy (7). Chronotherapy is an approach that strategically schedules drug administration according to the patient’s circadian rhythms to optimize the efficacy of cancer treatment (8–10). Previous studies on melanoma (11), non-small cell lung cancer (NSCLC) (12), and renal cell carcinoma (13) have shown that earlier time-of-day administration (ToDA) of ICIs is associated with better prognosis compared with later dosing. This suggests that time-of-day administration is a key factor influencing the efficacy of ICIs.

Although a prior single-center study suggested a potential effect of ToDA on nivolumab monotherapy in gastric cancer, the impact of ToDA on the prognosis of AGC patients receiving PD-1 inhibitor-based combination immunotherapy in a multicenter real-world setting remains unclear. Besides, studies are generally limited by single-center designs, small sample sizes, and lack of consensus on the optimal administration timing (14, 15). More importantly, no study to date has integrated ToDA with key clinical and pathological variables to construct a prognostic model for patients with AGC. Therefore, systematically evaluating the impact of PD-1 inhibitor infusion timing on AGC patients’ prognosis and elucidating its interplay with other clinical factors represents an important scientific question.

To bridge this gap, we conducted a multicenter retrospective study evaluating the association between ToDA and clinical outcomes of AGC patients receiving PD-1 inhibitor therapy. The study contributes to the field through three key advances (1): We identified 15:00 as the optimal cutoff value for ToDA and found that ToDA was an independent prognostic factor for progression-free survival (PFS) and overall survival (OS) (2). We established novel risk score models that, for the first time, integrate ToDA with key clinicopathological variables, effectively stratifying patients into distinct prognostic subgroups (3). We performed comprehensive subgroup analyses that revealed heterogeneity in the treatment effect of ToDA across patient subgroups with different clinical characteristics. In summary, this study establishes ToDA as a novel and important prognostic factor, providing evidence for exploring time-optimized immunotherapy in patients with AGC.

## 2 Materials and methods

### 2.1 Patient characteristics and data collection

A total of 416 AGC patients receiving PD-1 inhibitor treatment were enrolled in this study from three medical centers: 371 cases from the First Affiliated Hospital of Anhui Medical University, 40 cases from the Second Affiliated Hospital of Anhui Medical University, and 5 cases from the Second Affiliated Hospital of Bengbu Medical University. Clinical information was collected from the electronic record system, which included gender, age, the Eastern Cooperative Oncology Group Performance Status (ECOG PS), metastasis to the peritoneum, liver, and lung, treatment lines, human epidermal growth factor receptor 2 (HER-2) status, programmed cell death ligand 1 (PD-L1), neutrophil-to-lymphocyte ratio (NLR), and body mass index (BMI). Based on previous studies (8, 16), continuous variables were categorized using clinically meaningful cutoff thresholds (age: 65 years, NLR: 5, BMI: 25). This study was approved by the Ethics Committee of the First Affiliated Hospital of Anhui Medical University (PJ 2024-06-37).

### 2.2 Inclusion and exclusion criteria

The inclusion criteria: (1) Patients with gastric cancer verified by pathology. (2) Patients with AGC that is at stage IV or unresectable. (3) Patients who had received at least two cycles of PD-1 inhibitor combination therapy. (4) Patients with complete laboratory and clinical data. The exclusion criteria: (1) Patients with incomplete medical records or substantial gaps in clinical information. (2) Patients with active autoimmune or infectious disorders at enrollment. (3) Patients with a history of another malignancy within the past five years. (4) Patients with incomplete follow-up data.

### 2.3 Study design

We collected data on the timing of patients’ first three cycles of PD-1 inhibitor therapy. Following previous studies (17), patients in the early ToDA group received PD-1 inhibitor therapy before cutoff time, whereas those in the late ToDA group had at least one course of therapy administered after cutoff time. Based on previous research reports (12, 18, 19) and sensitivity analyses of various time windows within this cohort, we iteratively tested time points at 30-minute intervals between 11:00 and 16:30. The optimal cutoff time was selected as the time point at which the hazard ratios (HRs) for PFS and OS were most significant across the entire study cohort. To evaluate the stability of the cutoff, we performed an internal validation using bootstrap resampling with 1,000 iterations.

Sintilimab (200 mg q3w), Nivolumab (240 mg q2w), Pembrolizumab (200 mg q3w), Camrelizumab (200 mg q3w), or other PD-1 inhibitors were used until disease progression, death or the occurrence of an intolerable adverse event. In accordance with the clinical guidelines, immunotherapy was used in combination with chemotherapy and targeted therapy. The primary endpoint was PFS, defined as the time from the first administration of immunotherapy until disease progression, death, or a censoring event. OS was calculated from the first treatment with PD-1 inhibitors to death or last follow-up.

### 2.4 Statistical analysis

Categorical variables were expressed as frequencies and percentages. The 1: 2 optimal pair matching was performed by ‘MatchIt’ package with the objective of minimizing the sum of absolute pairwise distances in the matched sample to balance the baseline characteristics and potential confounders. Balance was assessed using standardized mean difference (SMD) (20). Survival data were analyzed using Kaplan–Meier survival curves and Log–rank test. Multivariable Cox regression analysis was performed including variables with *P* < 0.1 in the univariable Cox regression analysis. Variables included in the Cox regression models satisfied the proportional hazards assumption (P > 0.05). Subgroup analyses and corresponding forest plots were generated for all covariates used in PSM to explore potential heterogeneity in treatment effects. Based on variables with P < 0.05 in the multivariable Cox regression analyses, we developed risk score models comprising independent prognostic factors in the overall cohort, with each risk factor assigned a score of 1. Using this score, we divided the patients into three subgroups: the low- (score 0-2), middle- (score 3), high-risk groups (score ≥ 4). A two-sided *P* < 0.05 was considered statistically significant. All statistical analyses were performed using the R software (version 4.2.2), along with the use of MSTATA software.

## 3 Results

### 3.1 Patient characteristics and study flow

This multicenter retrospective study identified 628 AGC patients receiving PD-1 inhibitor therapy between September 2019 and August 2025. The follow-up cutoff date is April 2026. In total, 212 patients were excluded due to missing clinical information or incomplete medical records (n = 160), acute infectious diseases or autoimmune disorders at enrollment (n = 3), a history of another malignancy within the past five years (n = 18), and incomplete follow-up data (n = 31). Ultimately, 416 AGC patients were enrolled in the subsequent analysis (**Figure 1**). Among 416 patients, the median age was 62 years (29–84 years), and 304 patients (73.1%) were male. Most patients (91.8%) had an ECOG PS of 0-1. Peritoneal metastases were detected in 74 patients (17.8%), liver metastasis in 201 patients (48.3%), and lung metastasis in 62 patients (14.9%). 301 patients (72.4%) received first-line PD-1 inhibitor therapy, and 41 patients (9.9%) were HER-2 positive. Additionally, PD-L1 expression status was evaluable in 116 patients. According to the research (4, 5), a PD-L1 CPS < 1 is considered negative, and ≥ 1 is considered positive. 32 patients were classified as PD-L1 status negative, and 84 patients were classified as PD-L1 status positive. Additionally, MSI or MMR status was assessed in 106 patients. MSI-L/MSS and pMMR were considered negative, while MSI-H and dMMR were considered positive. Among these, 100 patients were negative, and only 6 patients were positive. Because of the limited availability and extremely low number of positive cases, MSI/MMR status was not incorporated into the subsequent multivariable models. Patients received at least two courses of PD-1 inhibitor therapy combined with chemotherapy and/or targeted therapy. Additionally, 390 patients (93.8%) received at least three cycles of PD-1 inhibitor treatment.

**Figure 1:**
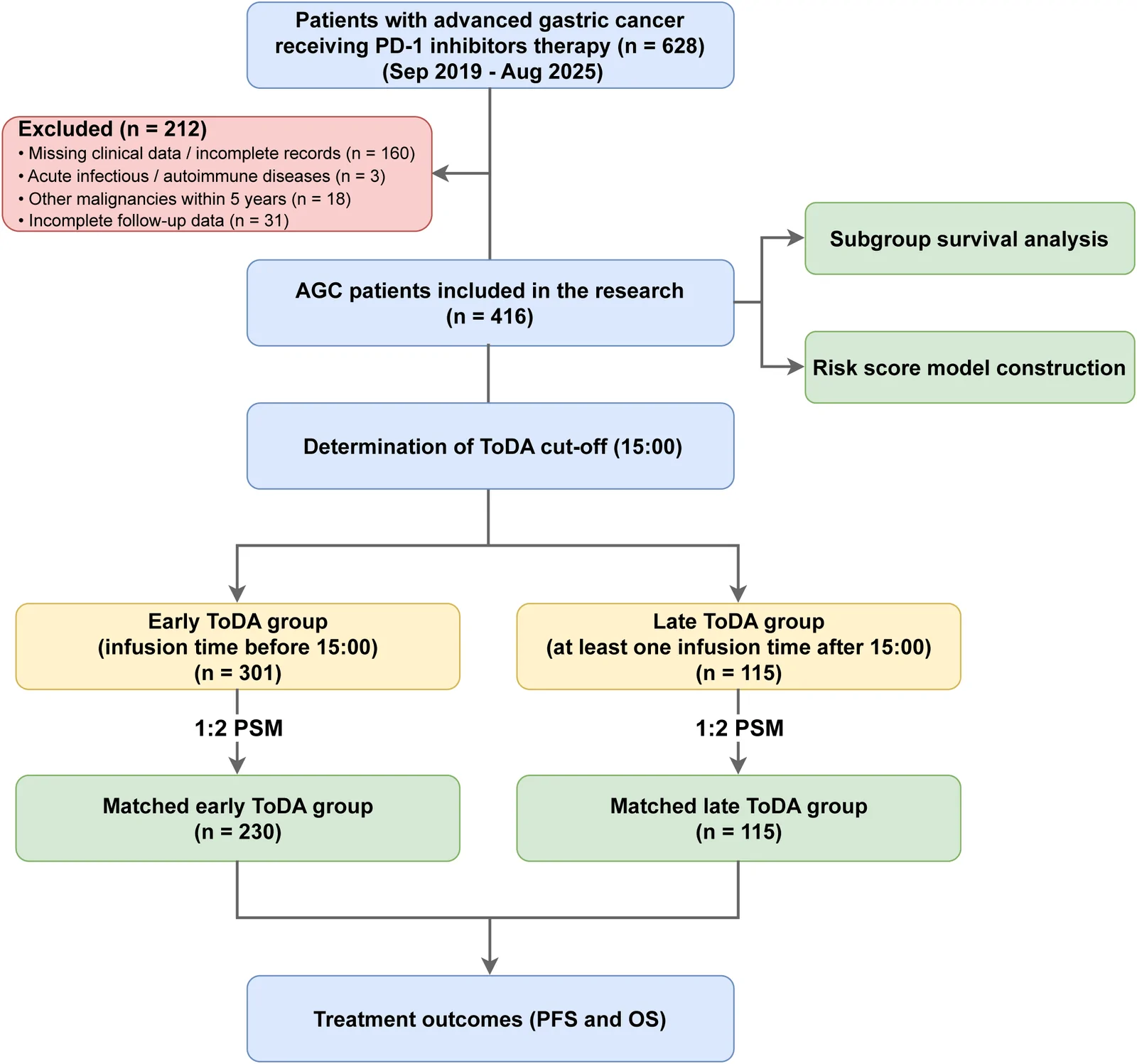
The flowchart of the study design and patient selection process. AGC, advanced gastric cancer; ToDA, time-of-day administration; PSM, propensity score matching; PFS, progression-free survival; OS, overall survival. Patients who did not meet the inclusion criteria were excluded.

**Table 1** presents characteristics before and after PSM. 301 patients were categorized into the early ToDA group (all infusion before 15:00), whereas 115 patients were assigned to the late ToDA group (at least one infusion time after 15:00). After 1:2 PSM, 115 patients from the late ToDA group were matched to 230 patients from the early ToDA group, resulting in a matched cohort of 345 patients. A notable reduction in SMD after matching can be observed (SMD < 0.1), indicating that the PSM effectively improved the balance of covariates between the two treatment groups.

**Table 1:** Baseline characteristics of AGC patients treated with PD-1 inhibitors.

Characteristics	Before PSM			After PSM		
	Early ToDA (N = 301)	Late ToDA (N = 115)	SMD^†^	Early ToDA (N = 230)	Late ToDA (N = 115)	SMD^†^
Gender, n (%)						
Male	219 (73%)	85 (74%)	0.026	168 (73%)	85 (74%)	0.020
Female	82 (27%)	30 (26%)	-0.026	62 (27%)	30 (26%)	-0.020
Age, n (%)						
≤65	167 (55%)	63 (55%)	-0.014	127 (55%)	63 (55%)	-0.009
>65	134 (45%)	52 (45%)	0.014	103 (45%)	52 (45%)	0.009
ECOG PS, n (%)						
≤1	275 (91%)	107 (93%)	0.066	216 (94%)	107 (93%)	-0.034
≥2	26 (9%)	8 (7%)	-0.066	14 (6%)	8 (7%)	0.034
Peritoneal metastasis, n (%)						
Negative	246 (82%)	96 (83%)	0.047	191 (83%)	96 (83%)	0.012
Positive	55 (18%)	19 (17%)	-0.047	39 (17%)	19 (17%)	-0.012
Liver metastasis, n (%)						
Negative	156 (52%)	59 (51%)	-0.010	124 (54%)	59 (51%)	-0.052
Positive	145 (48%)	56 (49%)	0.010	106 (46%)	56 (49%)	0.052
Lung metastasis, n (%)						
Negative	256 (85%)	98 (85%)	0.005	195 (85%)	98 (85%)	0.012
Positive	45 (15%)	17 (15%)	-0.005	35 (15%)	17 (15%)	-0.012
Treatment lines, n (%)						
1	216 (72%)	85 (74%)	0.049	168 (73%)	85 (74%)	0.020
≥2	85 (28%)	30 (26%)	-0.049	62 (27%)	30 (26%)	-0.020
HER-2 status, n (%)						
Negative/unknown	271 (90%)	104 (90%)	0.014	205 (89%)	104 (90%)	0.044
Positive	30 (10%)	11 (10%)	-0.014	25 (11%)	11 (10%)	-0.044
PD-L1 status, n (%)						
Negative/unknown	238 (79%)	94 (82%)	0.069	190 (83%)	94 (82%)	-0.023
Positive	63 (21%)	21 (18%)	-0.069	40 (17%)	21 (18%)	0.023
NLR, n (%)						
≤5	258 (86%)	108 (94%)	0.343	216 (94%)	108 (94%)	<0.001
>5	43 (14%)	7 (6%)	-0.343	14 (6%)	7 (6%)	<0.001
BMI, n (%)						
≤25	259 (86%)	104 (90%)	0.149	208 (90%)	104 (90%)	<0.001
>25	42 (14%)	11 (10%)	-0.149	22 (10%)	11 (10%)	<0.001

^†^ SMD = Standardized Mean Difference (SMD <0.1 was considered acceptable balance).

AGC, advanced gastric cancer; ToDA, time-of-day administration; PSM, propensity score matching; ECOG PS, Eastern Cooperative Oncology Group performance status; PD**-**L1, programmed cell-ligand 1; NLR, neutrophil-to-lymphocyte ratio; BMI, body mass index.

### 3.2 ToDA threshold determination for distinguishing survival outcomes

To investigate how the timing of PD-1 inhibitor administration affects the prognosis of AGC patients, we compared the differences in PFS and OS between patients treated before versus after consecutive 30-minute time windows spanning 11:00 to 16:30, and we displayed the prognostic risks using forest plots. The results showed that the most pronounced differences in both PFS and OS were observed when 15:00 was used as the cutoff point, suggesting that 15:00 is the optimal threshold for predicting outcomes following PD-1 therapy (**Figure 2**). Compared with patients who received PD-1 inhibitor after 15:00, those who received treatment before 15:00 had longer PFS and OS. In the bootstrap validation, 15:00 was selected as the most significant cutoff in 64.7% of the replicates for PFS and in 46.2% for OS. The 95% confidence interval (CI) for the hazard ratio (HR) at 15:00 remained significant for both PFS (HR = 1.423, 95% CI: 1.138–1.822) and OS (HR = 1.496, 95% CI: 1.092–2.029). These findings support the stability of the 15:00 cutoff, while prospective validation remains necessary. Consequently, 15:00 was set as the cutoff time for dividing patients into early and late ToDA groups, as this time point yielded the most significant HR for PFS and OS. This cutoff selection is data-driven and requires future validation.

**Figure 2:**
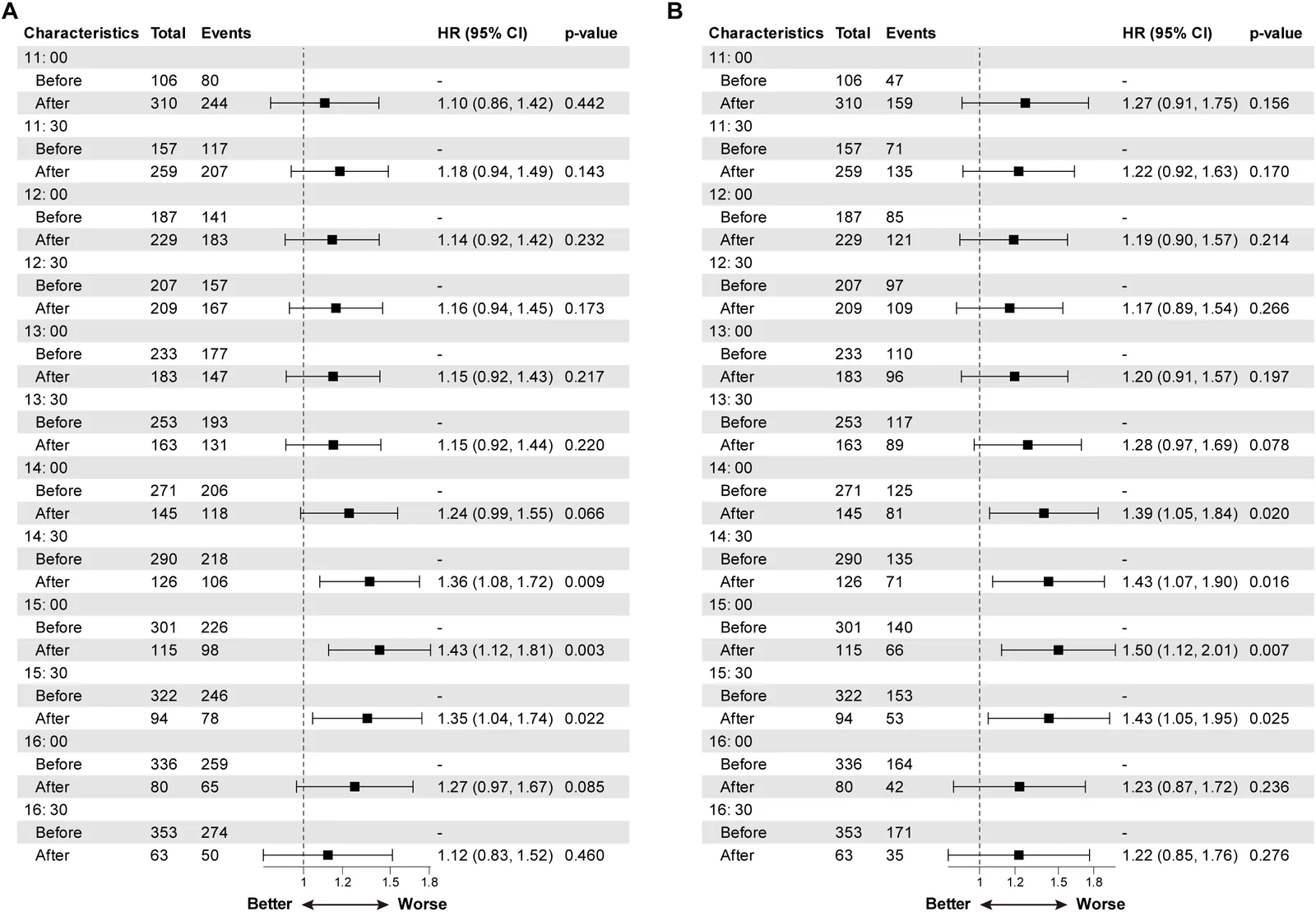
Sensitivity analysis to determine the optimal ToDA cutoff time. Forest plots display the HRs, 95% CIs, and p-values for PFS **(A)** and OS **(B)** across consecutive 30-minute time windows from 11:00 to 16:30 (Time labels are shown as 24-hour clock time in hours).

In addition, to address potential ToDA classification bias, we performed a sensitivity analysis (**Supplementary Table 1**). 301 patients completed all three infusions before 15:00, 89 patients completed one infusion after 15:00, 23 patients completed two infusions after 15:00, and 3 patients completed all three infusions after 15:00. Even among patients who had only one infusion after 15:00, PFS (HR = 1.473, 95% CI: 1.135–1.912, P = 0.004) and OS (HR = 1.466, 95% CI: 1.064–2.021, P = 0.019) were significantly worse. For patients who received two or three infusions after 15:00, the point estimates of the HR were similarly elevated, but these associations did not reach statistical significance (PFS: HR = 1.216, 95% CI: 0.760–1.944, P = 0.415, HR = 2.049, 95% CI: 0.654–6.415, P = 0.218; OS: HR = 1.554, 95% CI: 0.879–2.746, P = 0.130, HR = 2.450, 95% CI: 0.605–9.920, P = 0.209). Given the small sample sizes in these subgroups, in the exploratory analysis of this study, we used at least one infusion as the definition of late ToDA. However, this definition requires further validation in the future.

### 3.3 Prognostic value of ToDA in AGC patients

To evaluate the independent impact of PD-1 inhibitor administration timing on both PFS and OS in AGC patients after PSM, we performed univariable and multivariable Cox analyses. For PFS, the multivariable results identified peritoneal metastasis (HR = 1.455, 95% CI: 1.050–2.015, *P* = 0.024), liver metastasis (HR = 1.585, 95% CI: 1.223–2.054, *P* < 0.001), treatment lines ≥ 2 (HR = 2.146, 95% CI: 1.638–2.812, *P* < 0.001), and the late ToDA group (HR = 1.536, 95% CI: 1.195–1.976, *P* < 0.001) as independent adverse prognostic factors, whereas HER-2 positivity (HR = 0.458, 95% CI: 0.292–0.720, *P* < 0.001) and PD-L1 positivity (HR = 0.624, 95% CI: 0.438–0.890, *P* = 0.009) emerged as independent protective factors (**Table 2**). For OS, the multivariable analysis revealed that treatment lines ≥ 2 (HR = 2.227, 95% CI: 1.621–3.059, *P* < 0.001), high NLR level (HR = 1.908, 95% CI: 1.060–3.437, *P* = 0.031), and the late ToDA group (HR = 1.591, 95% CI: 1.165–2.173, *P* = 0.004) were independent adverse prognostic factors, meanwhile HER-2 positivity (HR = 0.541, 95% CI: 0.315–0.930, *P* = 0.026), PD-L1 positivity (HR = 0.536, 95% CI: 0.327–0.880, *P* = 0.014), and high BMI level (HR = 0.496, 95% CI: 0.261–0.944, *P* = 0.033) served as independent protective factors (**Table 3**). Of note, multivariable Cox regression analyses conducted across the overall cohort (before PSM) revealed that the independent prognostic factors were largely consistent for both PFS and OS and significance of ToDA remained consistent (**Figures 3**, **4**). Additionally, the multivariable Cox analyses in **Tables 2**, **3** were performed after PSM to explore the prognostic effect of ToDA while minimizing confounding. In contrast, the risk score models were developed in the overall cohort to preserve the full sample size and the natural distribution of all clinical covariates, thereby integrating multiple prognostic factors for comprehensive risk stratification. Taken together, these results suggest that administering PD-1 inhibitor therapy after the optimal cutoff time (15:00) is significantly associated with worse PFS and OS.

**Table 2:** Cox regression analysis of PFS in AGC patients treated with PD-1 inhibitors after propensity score matching.

Characteristics	Univariable		Multivariable	
	HR (95% CI)	P value	HR (95% CI)	P value
Gender				
Male	1 (Reference)			
Female	0.845 (0.641-1.114)	0.233	–	–
Age				
≤65	1 (Reference)			
>65	0.941 (0.739-1.198)	0.622	–	–
ECOG PS				
≤1	1 (Reference)			
≥2	1.113 (0.696-1.777)	0.655	–	–
Peritoneal metastasis				
Negative	1 (Reference)			
Positive	1.430 (1.056-1.938)	0.021	1.455 (1.050-2.015)	**0.024**
Liver metastasis				
Negative	1 (Reference)			
Positive	1.257 (0.989-1.596)	0.061	1.585 (1.223-2.054)	**<0.001**
Lung metastasis				
Negative	1 (Reference)			
Positive	1.065 (0.761-1.491)	0.714	–	–
Treatment lines				
1	1 (Reference)			
≥2	2.098 (1.609-2.737)	<0.001	2.146 (1.638-2.812)	**<0.001**
HER-2 status				
Negative/unknown	1 (Reference)			
Positive	0.512 (0.329-0.797)	0.003	0.458 (0.292-0.720)	**<0.001**
PD-L1 status				
Negative/unknown	1 (Reference)			
Positive	0.599 (0.423-0.848)	0.004	0.624 (0.438-0.890)	**0.009**
ToDA group				
Early ToDA group	1 (Reference)			
Late ToDA group	1.463 (1.140-1.877)	0.003	1.536 (1.195-1.976)	**<0.001**
NLR level				
≤5	1 (Reference)			
>5	1.158 (0.707-1.895)	0.560	–	–
BMI level				
≤25	1 (Reference)			
>25	0.847 (0.565-1.271)	0.423	–	–

AGC, advanced gastric cancer; PFS, progression-free survival; ECOG PS, Eastern Cooperative Oncology Group performance status; ToDA, time-of-day administration; PD**-**L1, programmed cell-ligand 1; NLR, neutrophil-to-lymphocyte ratio; BMI, body mass index.

N = 345; N events = 271; C-index = 0.630; AIC = 2,675; the likelihood ratio test was significant.

Values in bold indicate those that are statistically significant in the multivariate Cox regression analysis.

**Table 3:** Cox regression analysis of OS in AGC patients treated with PD-1 inhibitors after propensity score matching.

Characteristics	Univariable		Multivariable	
	HR (95% CI)	P value	HR (95% CI)	P value
Gender				
Male	1 (Reference)			
Female	0.859 (0.610-1.209)	0.383	–	–
Age				
≤65	1 (Reference)			
>65	1.270 (0.939-1.716)	0.121	–	–
ECOG PS				
≤1	1 (Reference)			
≥2	1.340 (0.744-2.410)	0.329	–	–
Peritoneal metastasis				
Negative	1 (Reference)			
Positive	1.444 (0.982-2.124)	0.062	1.256 (0.844-1.869)	0.261
Liver metastasis				
Negative	1 (Reference)			
Positive	1.216 (0.899-1.646)	0.204	–	–
Lung metastasis				
Negative	1 (Reference)			
Positive	0.883 (0.574-1.360)	0.573	–	–
Treatment lines				
1	1 (Reference)			
≥2	2.290 (1.676-3.129)	<0.001	2.227 (1.621-3.059)	**<0.001**
HER-2 status				
Negative/unknown	1 (Reference)			
Positive	0.567 (0.333-0.964)	0.036	0.541 (0.315-0.930)	**0.026**
PD-L1 status				
Negative/unknown	1 (Reference)			
Positive	0.511 (0.313-0.833)	0.007	0.536 (0.327-0.880)	**0.014**
ToDA group				
Early ToDA group	1 (Reference)			
Late ToDA group	1.590 (1.167-2.166)	0.003	1.591 (1.165-2.173)	**0.004**
NLR level				
≤5	1 (Reference)			
>5	1.918 (1.087-3.383)	0.025	1.908 (1.060-3.437)	**0.031**
BMI level				
≤25	1 (Reference)			
>25	0.478 (0.252-0.906)	0.024	0.496 (0.261-0.944)	**0.033**

AGC, advanced gastric cancer; OS, overall survival; ECOG PS, Eastern Cooperative Oncology Group performance status; ToDA, time-of-day administration; PD**-**L1, programmed cell-ligand 1; NLR, neutrophil-to-lymphocyte ratio; BMI, body mass index.

N = 345; N events = 170; C-index = 0.683; AIC = 1,735; the likelihood ratio test was significant.

Values in bold indicate those that are statistically significant in the multivariate Cox regression analysis.

**Figure 3:**
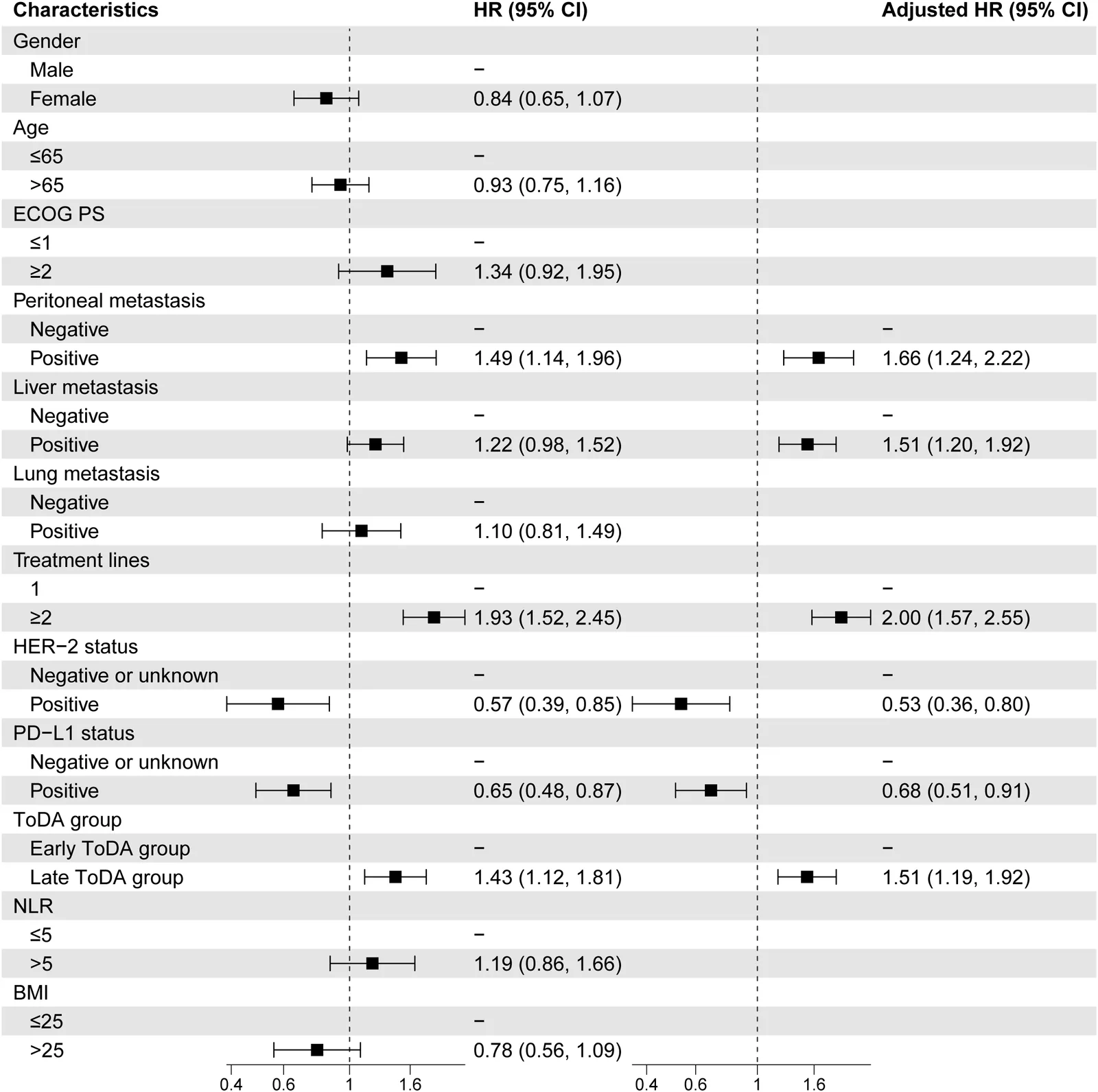
Forest plot detailing the univariable and multivariable Cox regression analyses identifying independent prognostic factors for PFS in the overall cohort before PSM. HR, hazard ratio; CI, confidence interval; ECOG, Eastern Cooperative Oncology Group; ToDA, time-of-day administration; PD-L1, programmed cell-ligand 1; NLR, neutrophil-to-lymphocyte ratio; BMI, body mass index. N = 416; N events = 324; C-index = 0.627; AIC = 3,333; the likelihood ratio test was significant.

**Figure 4:**
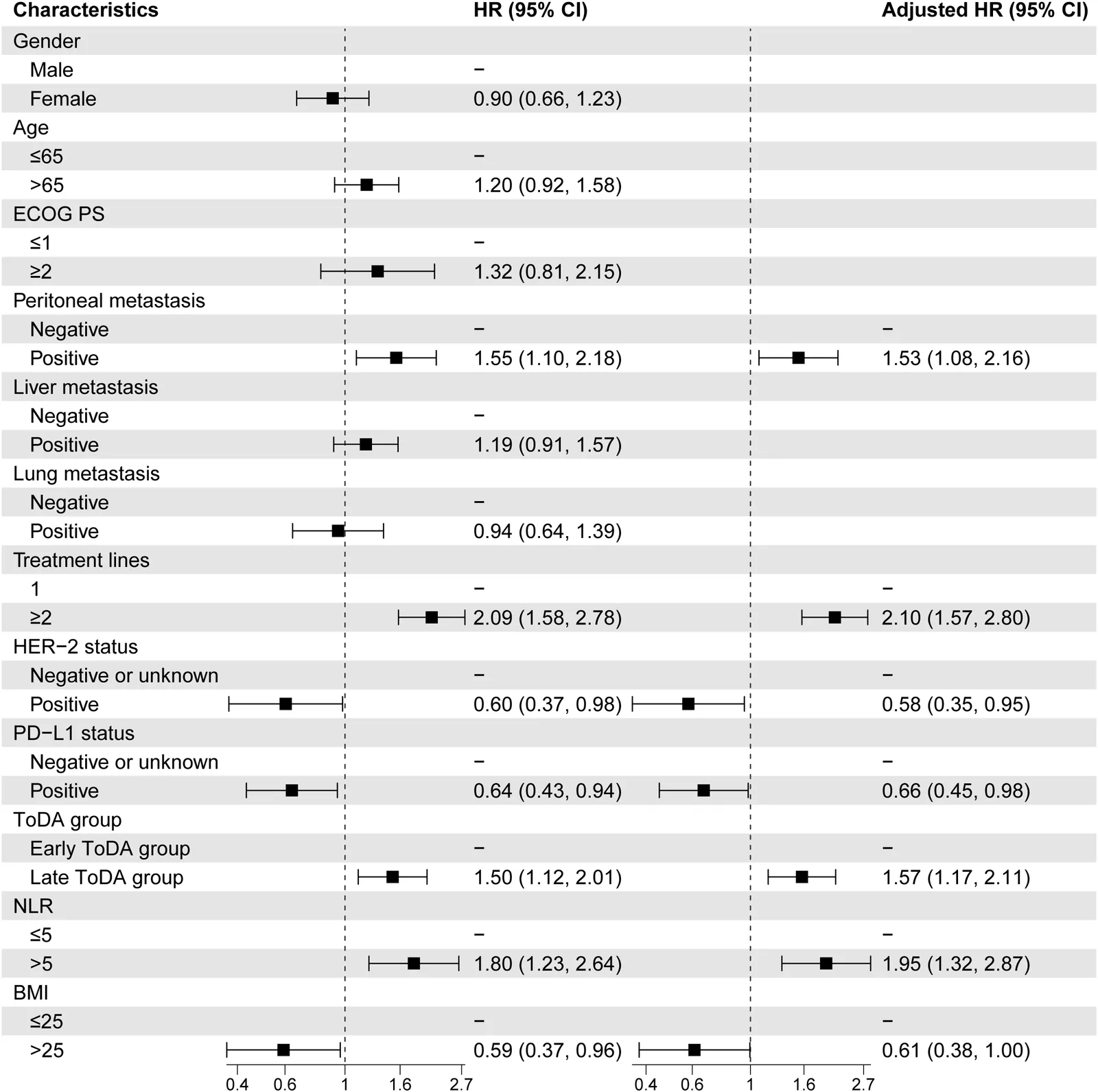
Forest plot detailing the univariable and multivariable Cox regression analyses identifying independent prognostic factors for OS in the overall cohort before PSM. HR, hazard ratio; CI, confidence interval; ECOG, Eastern Cooperative Oncology Group; ToDA, time-of-day administration; PD-L1, programmed cell-ligand 1; NLR, neutrophil-to-lymphocyte ratio; BMI, body mass index. N = 416; N events = 206; C-index = 0.679; AIC = 2,179; the likelihood ratio test was significant.

### 3.4 Kaplan–Meier survival curves and risk score model

Following PSM, the early ToDA group had significantly longer median PFS than the late ToDA group (mPFS, 8.6 months, 95% CI: 7.141–10.139, vs. 6.0 months, 95% CI: 4.746–7.154, *P* = 0.003; **Figure 5A**). Similarly, the median OS in the early ToDA group remained significantly better than the late ToDA group (mOS, 23.2 months, 95% CI: 18.305–28.015, vs. 14.3 months, 95% CI: 9.822–18.758, *P* = 0.003; **Figure 5B**). Based on the results of the Cox regression analysis in the overall cohort described above, we incorporated independent prognostic factors associated with PFS and OS into the risk score models. Factors associated with PFS included peritoneal metastasis, liver metastasis, treatment lines, HER-2 status, PD-L1 status, and ToDA group (**Figure 3**). Factors associated with OS included peritoneal metastasis, treatment lines, HER-2 status, PD-L1 status, NLR levels, and ToDA group (**Figure 4**). Specifically, late ToDA group, peritoneal metastasis positive, liver metastasis positive, negative HER-2 status, negative PD-L1 status, NLR > 5, and two or more prior lines of treatment, which were negatively correlated with PFS or OS, were recorded as a score of 1, and their corresponding opposites were scored as 0. Based on the total scores for each factor, we divided the patients into three subgroups in the overall cohort. Low-risk (scores 0-2), middle-risk (scores 3) and high-risk (scores ≥ 4). For PFS, the low-risk group had the longest mPFS of 11.5 months (95% CI: 8.154–14.906), followed by the middle-risk group of 7.0 months (95% CI: 5.874–8.126), and the high-risk group of only 4.7 months (95% CI: 3.774–5.626, *P* < 0.001; **Figure 5C**). Similarly, the prognostic score model for OS showed consistent results, with the low-risk group having the longest mOS at 28.7 months (95% CI: 24.381–32.919), followed by the middle-risk group at 14.6 months (95% CI: 10.561–18.539), and the high-risk group at only 10.2 months (95% CI: 7.601–12.699, *P* < 0.001; **Figure 5D**).

**Figure 5:**
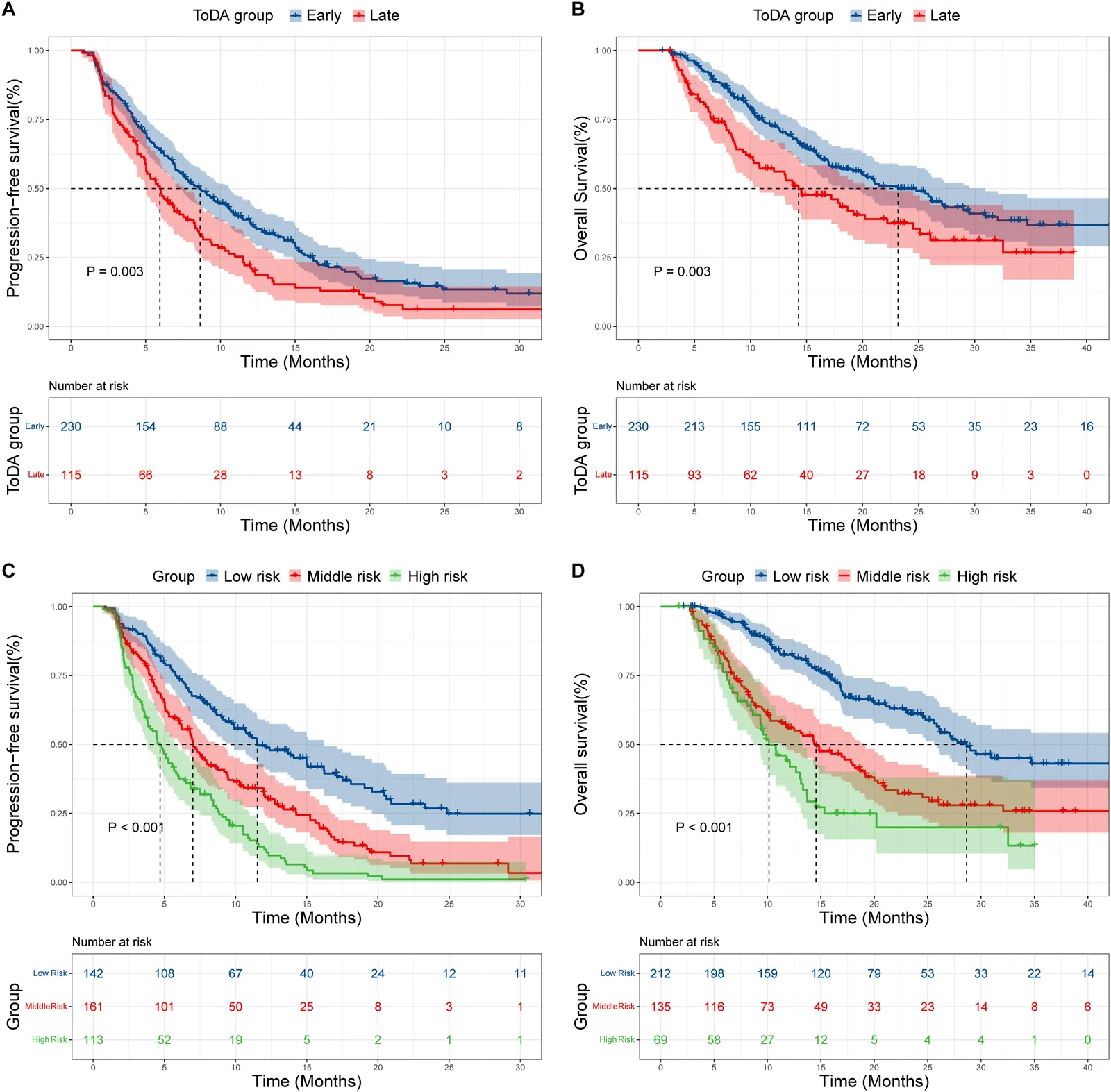
Kaplan–Meier survival curves for patients with advanced gastric cancer. **(A)** PFS and **(B)** OS compared between the early and late ToDA groups after PSM (N = 345). **(C)** PFS and **(D)** OS stratified by the prognostic risk score model, categorizing patients into low-risk, middle-risk, and high-risk groups in the overall cohort (N = 416). ToDA, time-of-day administration. Numbers at risk are shown below each panel.

### 3.5 Subgroup survival analysis

To further evaluate whether the prognostic impact of PD-1 inhibitor administration timing varied across baseline clinical subgroups, we performed subgroup analyses in the overall population. The early ToDA group had significantly longer PFS compared with the late ToDA group (HR = 1.427, 95% CI: 1.125–1.812, *P* = 0.003). Specifically, the effect remained significant in the subgroups of male patients (HR = 1.460, 95% CI: 1.108–1.922, *P* = 0.007), patients aged ≤ 65 years (HR = 1.607, 95% CI: 1.174–2.200, *P* = 0.003), patients with an ECOG PS ≤ 1 (HR = 1.490, 95% CI: 1.161–1.911, *P* = 0.002), patients without peritoneal metastasis (HR = 1.513, 95% CI: 1.159–1.973, *P* = 0.002), patients without lung metastasis (HR = 1.496, 95% CI: 1.156–1.937, *P* = 0.002), patients with negative or unknown status of HER-2 (HR = 1.474, 95% CI: 1.150–1.889, *P* = 0.002) or PD-L1 (HR = 1.427, 95% CI: 1.101–1.849, *P* = 0.007), and patients with low NLR (HR = 1.495, 95% CI: 1.164–1.920, *P* = 0.002) and low BMI (HR = 1.486, 95% CI: 1.156–1.911, *P* = 0.002) (**Figure 6**).

**Figure 6:**
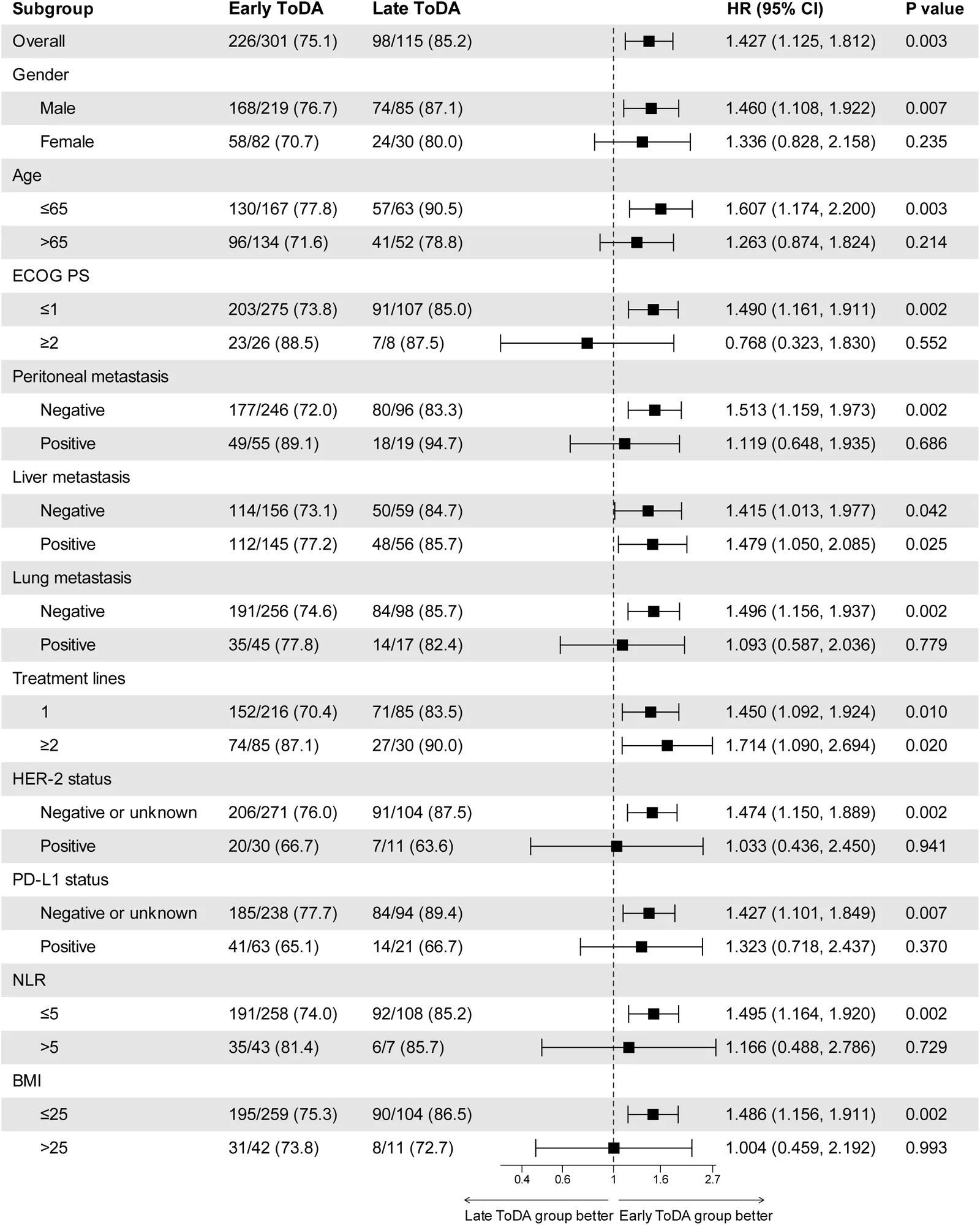
Subgroup analysis of PFS evaluating the impact of early versus late ToDA across various baseline clinicopathological characteristics in the overall cohort. HRs > 1 indicate better outcomes for the early ToDA group. HR, hazard ratio; CI, confidence interval; ECOG PS, Eastern Cooperative Oncology Group performance status; ToDA, time-of-day administration; PD-L1, programmed cell-ligand 1; NLR, neutrophil-to-lymphocyte ratio; BMI, body mass index.

Similarly, the early ToDA group was associated with significantly longer OS (HR = 1.501, 95% CI: 1.120–2.012, *P* = 0.007). Subgroup analyses revealed that this association was significant in males (HR = 1.532, 95% CI: 1.088–2.156, *P* = 0.015), patients aged ≤ 65 years (HR = 1.626, 95% CI: 1.088–2.431, *P* = 0.018), those with ECOG PS ≤ 1 (HR = 1.557, 95% CI: 1.148–2.111, *P* = 0.004), patients without peritoneal metastasis (HR = 1.659, 95% CI: 1.198–2.296, *P* = 0.002), those with negative or unknown HER-2 status (HR = 1.468, 95% CI: 1.080–1.995, *P* = 0.014), and negative or unknown PD-L1 status (HR = 1.590, 95% CI: 1.162–2.176, *P* = 0.004). The association was also significant in patients without lung metastasis (HR = 1.560, 95% CI: 1.137–2.141, *P* = 0.006). The association was particularly strong in patients who received ≥ 2 lines therapy (HR = 2.339, 95% CI: 1.467–3.730, *P* < 0.001) but not significant in those with first-line therapy. The effect was consistent across low NLR (HR = 1.719, 95% CI: 1.262–2.341, *P* = 0.001) and low BMI groups (HR = 1.558, 95% CI: 1.150–2.110, *P* = 0.004) (**Figure 7**).

**Figure 7:**
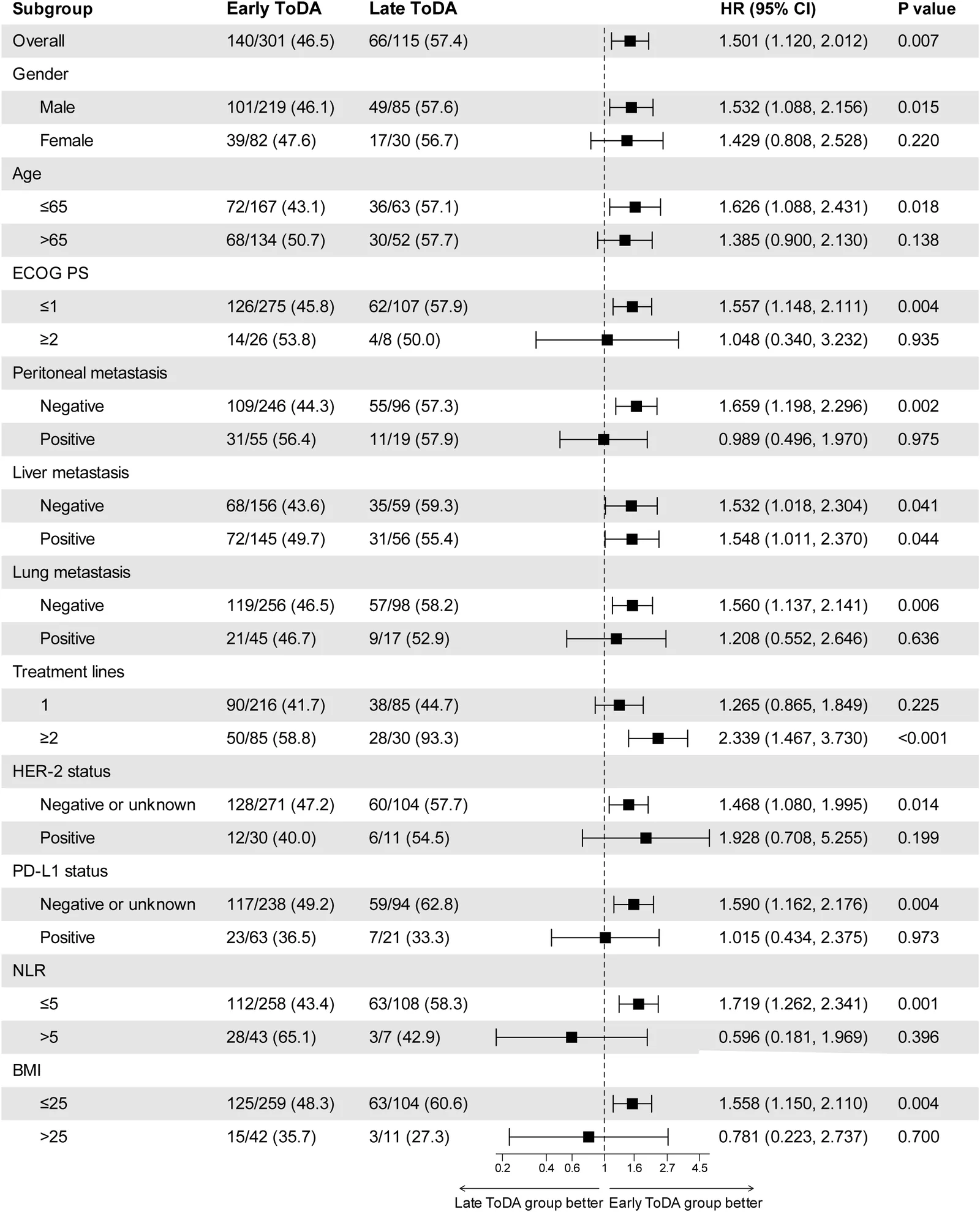
Subgroup analysis of OS evaluating the impact of early versus late ToDA across various baseline clinicopathological characteristics in the overall cohort. HRs > 1 indicate better outcomes for the early ToDA group. HR, hazard ratio; CI, confidence interval; ECOG PS, Eastern Cooperative Oncology Group performance status; ToDA, time-of-day administration; PD-L1, programmed cell-ligand 1; NLR, neutrophil-to-lymphocyte ratio; BMI, body mass index.

## 4 Discussion

Based on multicenter real-world data, this study provides evidence suggesting that earlier administration of PD-1 inhibitors (defined as before 15:00) is independently associated with PFS and OS. We further integrated the ToDA with key clinical and pathological characteristics to develop a preliminary risk stratification model for precision immunotherapy in gastric cancer. Subsequently, we evaluated differences in the prognostic impact of the ToDA across different subgroups.

Through sensitivity analysis, we identified 15:00 as the optimal cutoff time for distinguishing prognostic differences. Notably, given that the smaller proportion of patients in this cohort received PD-1 therapy after 15:00, and taking into account the distribution of the sample size and the results reported in a previous study (17), we defined the early ToDA group as patients who received PD-1 inhibitor therapy before 15:00, and the late ToDA group as patients who received at least one dose of PD-1 inhibitor therapy after 15:00. In prior studies of SCLC and melanoma, cutoff times of 15:00 and 16:00, respectively, were used to define early versus late administration (17, 19). Our results echo the core findings of these studies in gastric cancer, namely that early administration is superior to late administration. To address potential classification bias, we performed a sensitivity analysis. Even a single infusion after 15:00 was associated with significantly poorer survival compared to having all infusions before 15:00, while more frequent delays yielded elevated but non-significant risk estimates, likely due to very small subgroup sizes. These findings support the use of this definition in the present exploratory analysis. However, larger prospective studies are warranted to validate the definition of ToDA.

The biological mechanism may be related to the circadian rhythm of the human immune system. First, adaptive immune responses, particularly the proliferation and activation of T cells in peripheral blood and the tumor microenvironment, typically peak during the day (21, 22). Second, core circadian clock genes such as *BMAL1* and *CLOCK* intrinsically regulate antigen-presenting function in dendritic cells and subsequent T-cell cytotoxic activity, exhibiting circadian rhythmicity (23, 24). In addition, the expression of immune checkpoint molecules—such as PD-1 on lymphocytes and PD-L1 on tumor or myeloid cells—exhibits rhythmic fluctuations (25). Administering PD-1 inhibitors during the day aligns the pharmacological blockade of the PD-1/PD-L1 axis with the physiological peak periods of T-cell activation and tumor infiltration, thereby maximizing T-cell recognition and killing of tumor antigens (26, 27). However, these mechanisms are not yet clear and will need to be explored through further mechanistic research in the future. The variation in optimal cutoff times across cancer types may reflect heterogeneity in the circadian regulation of the tumor immune microenvironment, suggesting that specific analyses for different cancer types and patient populations are necessary when implementing time-of-day-based therapy in clinical practice.

Late infusion was found to be an independent risk factor for both PFS and OS in multivariable Cox analysis. These results remained robust after balancing potential confounding factors through PSM, indicating that infusion timing itself is a prognostic factor independent of traditional clinical and pathological characteristics. Our finding not only corroborates a recent small-scale study on nivolumab monotherapy for gastric cancer (14), but also further strengthens the reliability of this conclusion given the multicenter, large-sample nature of our study and the use of PSM to balance for confounding factors. Furthermore, we have combined ToDA with clinical and pathological characteristics to construct prognostic risk score models. Unlike previous studies that passively relied on baseline clinical and pathological characteristics and hematological indicators (28, 29), we incorporated the modifiable variable of ToDA into the model, thereby endowing it with the potential to actively guide interventions. Our model effectively classifies patients into different risk groups, providing a theoretical basis for the early identification of high-risk patients and precise chrono-immunotherapy in gastric cancer.

Subsequently, subgroup analyses revealed that the detrimental effect of late administration remained highly consistent across most major clinical subgroups, including patients aged ≤ 65 years, those with liver metastasis, and ECOG PS ≤ 1. This finding underscores the broad applicability of treatment timing as a prognostic factor and supports the potential for wider adoption of chronotherapy. However, no significant differences were observed in subgroups, such as women, patients older than 65 years, or those with peritoneal metastasis. This may be due to insufficient statistical power resulting from small subgroup sample sizes, or it may suggest heterogeneity in the circadian rhythm effect across distinct physiological states or tumor microenvironments, which requires clarification in future large-scale prospective studies.

The research has several limitations. First, despite the use of multicenter data and PSM, the retrospective design cannot completely exclude the influence of selection bias and residual confounding. The matched cohort may not fully represent the entire early ToDA population. Second, we did not collect data on dynamic circadian variations in immune cell subsets in peripheral blood or the tumor microenvironment, thus precluding direct mechanistic analysis of how circadian rhythms influence PD-1 inhibitor efficacy. Third, PD-L1 testing was performed only in a subset of patients; therefore, the possibility of selection bias due to missing PD-L1 data cannot be completely ruled out. Another limitation is that, although the bootstrap validation supports the stability of the 15:00 cutoff, this threshold was determined post hoc by maximal statistical separation. Independent validation in prospective cohorts is warranted to confirm the clinical relevance of this time point. Finally, the scoring model established in this study assigns equal weights to each factor, thereby ignoring differences in the magnitude of their effects, and has not been validated. Future research should validate this model in prospective randomized controlled studies.

Based on these findings, future prospective trials are needed to compare the impact of fixed early-infusion schedules versus late-time infusion on survival in AGC patients, thereby establishing the role of chronotherapy in GC treatment. Furthermore, dynamic collection of patient blood samples should be conducted to detect circadian fluctuations in clock gene expression and immune cell subsets, thereby revealing the optimal dosing windows and underlying mechanisms that precisely align with individual circadian phases. Finally, conducting prospective validation and optimization of the prognostic model we have developed will facilitate its translation into a clinical decision-support tool, ultimately enabling precise timing adjustments to further improve the efficacy of immunotherapy for gastric cancer.

## 5 Conclusions

In summary, this multicenter retrospective study identifies the timing of PD-1 inhibitor administration as a novel, independent, and modifiable prognostic factor for AGC patients. We established 15:00 as the optimal cutoff, demonstrating that infusions before this time significantly prolonged both PFS and OS. By integrating this ToDA factor with clinical-pathological variables, we developed risk-stratification models that effectively discriminate patients with distinct survival outcomes. Our findings provide compelling real-world evidence supporting the integration of chronotherapeutic strategies into the management of AGC. Future prospective trials are warranted to validate the survival benefit of early ToDA and to elucidate the underlying circadian biology.

## Data Availability

The raw data supporting the conclusions of this article will be made available by the authors, without undue reservation.
