# Supplementary material for: Early time-of-day infusion of PD-1 inhibitor improves prognosis in advanced gastric cancer: a multicenter real-world study
Source: Front Immunol. 2026 Sep 18;17:1945065. doi: 10.3389/fimmu.2026.1945065 (PMC13631753; doi:10.3389/fimmu.2026.1945065)
Supplement: Supplementary file 1 [file Table1.docx]

**Table S1** Sensitivity analysis of survival outcomes by number of late infusions

| **Number of**  **treatments after 15:00** | **PFS** |  | **OS** |  |
| --- | --- | --- | --- | --- |
|  | **HR (95% CI)** | **P value** | **HR (95% CI)** | **P value** |
| 0 | 1 (Reference) |  |  |  |
| 1 | 1.473 (1.135-1.912) | **0.004** | 1.466 (1.064-2.021) | **0.019** |
| 2 | 1.216 (0.760-1.944) | 0.415 | 1.554 (0.879-2.746) | 0.130 |
| 3 | 2.049 (0.654-6.415) | 0.218 | 2.450 (0.605-9.920) | 0.209 |
